# Equivalent End-Weakening Governing Crushing Modes and Crashworthiness of CFRP Tubes with Different End Triggers Under Quasi-Static Axial Compression

**DOI:** 10.3390/ma19194087

**Published:** 2026-09-24

**Authors:** Xi Li, Mengyan Zhu, Shixian Wu, Yazhi Li, Lei Yu

**Affiliations:** 1 School of Intelligent Welding Technology, Guangxi Technological College of Machinery and Electricity, Nanning 530007, China; 15529083200@163.com (X.L.); zhumengyan@gxcme.edu.cn (M.Z.); 2 School of Information and Communication, Guilin University of Electronic Technology, Guilin 541004, China; 3 School of Aeronautics, Northwestern Polytechnical University, Xi’an 710072, China; yazhi.li@nwpu.edu.cn; 4 China Aerospace Science and Technology Corporation Fourth Academy, Xi’an 710065, China; yulei198614@126.com

**Keywords:** CFRP tube, trigger, energy absorption, equivalent end-weakening

## Abstract

Quasi-static axial crushing tests were carried out on CFRP tubes with various end triggers (chamfer, tulip, crown, notch), aiming to reveal the joint regulation mechanism of geometric configuration and equivalent end-weakening on the crushing behavior of the tubes. Load–displacement responses, crashworthiness indicators and post-crushing failure morphologies were analyzed. Results indicate that the chamfer trigger can effectively reduce the initial peak load at the expense of mean crushing force and total energy absorption. Moderate tulip and crown triggers form cantilever petals and exhibit desirable two-stage crushing responses. However, excessive cutting intensifies the end-weakening effect, leading to simultaneous petal bending and matrix crushing and yielding a distinct unimodal response. Under the present test conditions, the notch trigger exhibits an inherent unimodal crushing characteristic. It delivers a high total energy absorption of up to 2186.2 J but suffers from a higher peak crushing load and lower crushing force efficiency, which reveals a clear performance trade-off. These observations demonstrate that geometric configuration and equivalent end-weakening exert a bidirectional coupled regulatory effect on crushing behavior. Furthermore, numerical simulation results show that the finite-element model can well predict the crushing load–displacement responses of tubes with different end modifications, while it fails to reproduce the typical petalling opening morphology of triggered specimens, which clarifies the application boundary of the simulation model. The results of this study can provide experimental references for the optimization design of end-triggered CFRP energy-absorbing tubes.

## 1. Introduction

Buffer energy-absorbing components are widely adopted in aerospace, automotive, and high-speed rail engineering fields [1,2,3,4,5,6]. Driven by lightweight and energy-saving requirements, composite materials have emerged as promising candidates for crash-energy-absorbing structures. Composite thin-walled tubes dissipate kinetic energy via multiple failure mechanisms, including fiber fracture, matrix cracking and inter-laminar delamination, and exhibit superior energy-absorption capacity compared with conventional metallic components that mainly rely on plastic deformation and buckling [7,8,9,10,11,12,13,14,15]. The crushing behavior of composite thin-walled structures is affected by multiple factors such as triggering configuration, laminate lay-up, cross-section geometry and loading velocity [7,8,9,10,11,12,13,14,15,16,17,18,19,20]. Among these factors, the end-triggering mechanism is of particular importance; it mitigates severe stress concentration at the onset of compression, avoids catastrophic brittle failure, and improves overall crashworthiness by initiating stable progressive failure [21,22,23,24,25,26,27,28,29,30,31,32,33,34,35,36,37].

Among various composite tubular configurations, circular-section thin-walled tubes combine favorable manufacturability and crashworthiness performance. They are widely utilized in crash-related components of railway and aerospace structures and serve as a common test carrier for end-trigger mechanism investigations [37,38,39,40,41,42,43,44,45,46,47,48,49,50,51,52,53,54,55,56]. Therefore, circular-section CFRP thin-walled tubes are adopted in this work. Previous studies focusing on end-triggers for circular tubes are reviewed below.

Considerable research efforts have been devoted to conventional end-trigger schemes, such as chamfer and tulip triggers, for circular composite tubes [21,26,54,55,56,57,58,59,60,61,62]. Huang et al. [26,57,58] examined flat-top, crown and 45°-bevel triggers and reported that combined crown-plus-bevel triggers improve crush-force efficiency. However, separate tests for the standalone crown trigger were absent in their test matrix, which prevents direct quantitative comparison between pure crown and chamfer triggers. Siromani et al. [21,59] investigated flat-end, 45°-bevel and external-cap triggers for cylindrical composite tubes. They found that both bevel and internal-cap triggers reduce peak force and elevate specific energy absorption; however, their discussion did not extend to notch-petal-based trigger forms. Song [60] studied chamfer triggers for circular composite tubes with different fiber orientations and concluded that single-ended chamfers reduce peak crushing force while maintaining high specific energy absorption, without exploring chamfer angle variations. Ma [61] compared inner, outer and double chamfers for [0/90]-laminated circular tubes and verified the performance improvement brought by chamfering, yet obvious regular trends against chamfer geometries were not observed within their test dataset. Huang et al. [26,57,58] also assessed ply-drop-off triggers and achieved moderate improvement in specific-energy absorption relative to non-triggered specimens. Song [62] compared petal-shaped triggers against flat-end specimens and confirmed the capability of petal triggers to decrease peak force and enhance energy absorption, though direct benchmarking against classical 45° chamfer triggers was missing. Palanivelu et al. [40,41,42,43,44,45] compared 45°-bevel and tulip triggers for circular composite shells; their tests suggested minor differences in specific-energy absorption while tulip triggers lowered peak crushing force by approximately 10%. Even so, the experimental setup did not support strict single-variable comparison for tulip trigger geometrical parameters.

With further development in this field, notched and petal-type triggers with end-cut features have attracted increasing attention. Cetin et al. [51] studied circular-hole triggering for Al/CFRP hybrid circular tubes. Based on Taguchi experimental design combined with the ARAS multi-criteria decision-making method, they assessed the effects of hole diameter, hole number and trigger position on crashworthiness indices. Their study concentrated on metal–composite hybrid systems, and pure CFRP tubes with chamfer and notch gradient trigger series were not covered. Baykasoğlu et al. [52] performed quasi-static crushing tests on notch-triggered GFRP circular tubes using a Taguchi L9 orthogonal-array design. They investigated three key parameters: notch diameter, circumferential notch quantity and notch-to-end distance, and further implemented multi-criteria optimization to balance specific energy absorption and peak crushing force. However, their experimental matrix was designed for orthogonal parametric screening, without focusing on continuous gradient variation of notch-related geometrical features. Padayachee [53] proposed a novel crown-tulip composite trigger for CFRP circular tubes under dynamic impact loading. By adding secondary crown cutouts between tulip petals, they attempted to remedy the low mean crush-force drawback of conventional tulip triggers. A calibrated numerical model was utilized for parametric scanning. Nevertheless, only a small number of physical specimens were manufactured for model calibration; gradient tests spanning from intact-wall chamfer triggers to heavily notched petal-type configurations were not conducted.

Although extensive investigations have been conducted on the axial crushing behavior of end-triggered CFRP energy-absorbing tubes, most existing studies focus on the performance of only one or a small number of trigger configurations. Systematic comparative research covering chamfer, tulip, crown and notch end-weakening geometries remains limited. In fact, end triggers alter not only the geometric profile but also introduce varying degrees of equivalent end-weakening effects. Nevertheless, the coupled regulatory mechanism of geometric configuration and equivalent end-weakening degree on crushing modes, load responses and crashworthiness performance has not been clearly elucidated. The combined effect of these two factors lacks systematic interpretation, which hinders the integrated optimal design of various end-triggered structures.

On this basis, CFRP thin-walled tubes with four typical end-triggered configurations (chamfer, tulip, crown and notch) are designed, and quasi-static axial crushing tests are carried out. By comparing the load–displacement responses, crashworthiness indicators and post-crushing failure morphologies of different trigger types, combined with DIC full-field measurement and visual recognition post-processing to track the dynamic evolution of high-strain regions frame by frame, this paper systematically reveals the joint action mechanism of geometric features and equivalent end-weakening on tube crushing behavior. Meanwhile, finite-element simulations are performed to evaluate the predictive capability of the numerical model for specimens with different end modifications and define its applicable range. The findings can provide experimental support and theoretical references for the selection and optimization design of end-triggered CFRP energy-absorbing structures.

## 2. Experimental Process

### 2.1. Specimen Design and Manufacturing

Single-layer laminates were fabricated using the 2126B medium-temperature-curing epoxy resin system supplied by Weihai Lanke Composite Materials Technology Co., Ltd. (Weihai, China) and Tenax UTS50 carbon fibers supplied by Teijin Limited (Tokyo, Japan). The fiber volume fraction of the composite laminates was approximately 63%, with a fiber areal density of 100 g/m^2^, and the single-layer thickness was controlled at 0.125 mm. The fabrication was performed under vacuum pressure at a heating rate of 5 °C/min. Specifically, the laminates were cured at 110 °C for 30 min and subsequently at 130 °C for another 30 min, followed by natural cooling under pressure to obtain single-layer prepreg sheets.

Based on the qualified single-layer prepregs, thin-walled cylindrical components were manufactured by winding and stacking the prepregs onto a 50 mm-diameter aluminum alloy mandrel, supplied by Shanghai Xinda Hengzhong Industrial Co., Ltd. (Shanghai, China), with a stacking sequence of [+45/−45/90/0/0/90/0]_s_. The lap width of each prepreg layer was approximately 5 mm, and the lap joints were evenly distributed at 90° intervals. During the winding process, the forming procedure was carried out under vacuum pressure with the same curing regime: heating at 5 °C/min to 110 °C for 30 min and 130 °C for 30 min, followed by natural cooling under pressure. The preparation process is illustrated in Figure 1.

The as-fabricated cylindrical specimens featured a circular cross-section, with a total length of 100 mm, an inner diameter of 50 mm, and a wall thickness of 2 mm.

A total of 4 different specimens with various end triggers (chamfer, tulip, crown, notch) were designed, as detailed in Figure 2 and Table 1, covering 36 distinct configurations. For statistical reliability, two replicate specimens were prepared for each configuration, yielding a total of 72 specimens. The dimensions of the tulip, crown and notch triggers are illustrated in Figure 2c, and their corresponding equivalent end-weakening geometric removal volumes (*Veq*) are listed in Table 2.

The machining process for end weakening is described as follows: A dedicated internal support fixture was adopted for clamping. Mastercam 2022 software was utilized for machining programming. A 6-mm-diameter solid carbide milling cutter (Shandong Chenbang CNC Equipment Co., Ltd., Weifang, China) dedicated to CFRP (two-flute, uncoated chip grooves, designed to prevent carbon powder accumulation and edge chipping) was selected for the finish machining of outer cylindrical surfaces, with a streamlined finishing strategy. The spindle speed was 6000 r/min, the feed rate was 2000 mm/min, and the cutting step was 0.15 mm. A 2 mm diameter solid carbide end mill (fine-edged finishing cutter) (Shandong Chenbang CNC Equipment Co., Ltd., Weifang, China) was used for machining axial grooves on the tube end, adopting the profile layer milling strategy. The spindle speed was 7000 r/min, the feed rate was 1000 mm/min, and the cutting depth per layer was 0.2 mm. The machining tolerance for all specimens was ±0.05 mm.

### 2.2. Test Setup and Process

#### 2.2.1. Lamina Testing and Material Parameters

To characterize the fundamental mechanical properties of the material, mechanical property tests were conducted in accordance with ASTM D3039 [63] (tension), ASTM D3518 [64] (shear), and ASTM D6641 [65] (compression) standards. Three replicate specimens were tested for each test group. The test setup is shown in Figure 3, and the corresponding material properties of the unidirectional laminates are listed in Table 3.

#### 2.2.2. Quasi-Static Axial Crushing Tests of Specimens

The typical experimental setup for the axial compression testing of the composite tube is shown in Figure 4. All tests were conducted under quasi-static axial compression conditions. All axial compression tests were performed on a Zwick Roell testing machine, ZwickRoell GmbH & Co., KG, Ulm, Germany, with a loading capacity of 100 kN at a cross-head speed of 5 mm/min (0.197 in/min). The nominal force measurement accuracy of the machine is ±1% FS, and the nominal displacement measurement accuracy is ±0.5%. The data acquisition frequency of the testing machine is 45 Hz.

A maximum crosshead displacement of 50 mm was applied to all specimens. This displacement range was selected to ensure adequate deformation data while avoiding excessive structural failure. In addition, corresponding load–displacement curves were recorded during the crushing process. Based on these data, the energy absorption characteristics of the 100 mm long specimens were determined.

A digital image correlation (DIC) system (an ARAMIS 4M system from GOM, Braunschweig, Germany) was used to capture the full-field deformation and strain fields during loading. The system employs two charge-coupled-device (CCD) cameras with a resolution of 2352 × 1728 pixels (4 megapixels). Sub-pixel interpolation provides a sub-pixel accuracy of 0.01 pixels. The measurable strain range spans from 0.01% to over 100%. The data acquisition frequency of the DIC is 1 Hz. The practical displacement measurement accuracy is dependent on the calibrated field of view.

### 2.3. Crashworthiness Assessment Parameters

In this manuscript, the following indicators are adopted to quantify the crushing energy absorption performance of thin-walled structures under axial loads, namely EA, Pm, PCF, CFE, SEA. All of these parameters can be obtained from the crushing load–displacement curves.

(1) The energy absorption (EA) is used to measure the ability of thin-walled structures to absorb energy during the crushing process. The load–displacement curve recorded through experiments is integrated to obtain:

(1) $$EA=\int_{0}^{d}F(x)dx$$

Here, d represents the crushing distance and F(x) is the load of axial compression.

(2) The average crushing load $P_{m}$ is used to measure the energy absorption per unit length of the failed crushed part:

(2) $$P_{m}=\frac{1}{d}\int_{0}^{d}F(x)dx$$

The greater the average crushing load, the stronger the energy absorption capacity of the structure under a certain specific deformation mode.

(3) The peak crushing force (PCF) can be directly obtained from the load–displacement curve. The peak crushing force is an important indicator for measuring the energy absorption characteristics of energy-absorbing components. An excessively high peak crushing force can threaten personnel safety. Therefore, in many optimization problems [20,30,41,66,67], the set target of the initial peak is relatively low.

(4) The crushing force efficiency (CFE) is defined as the ratio of the average crushing load to the initial peak load:

(3) $$CFE=\frac{P_{m}}{PCF}$$

The stability of the load during the crushing process is measured. The closer the ratio is to 1, the smaller the load fluctuation during the crushing process and the more stable the structural crushing.

(5) Specific energy absorption (SEA) is employed to assess the energy absorption capacity of thin-walled structures with various failure trigger mechanisms, quantifying the energy absorbed per unit mass of the structure, which is defined by:

(4) $$SEA=\frac{EA(\delta )}{m}=\frac{EA(\delta )}{\rho A\delta }$$

Here, δ represents the compaction distance, m is the mass of the thin-walled structure, ρ is the material density, and A is the effective cross-sectional area. The greater the specific energy absorption, the better the energy absorption effect of the structure.

### 2.4. Finite-Element Modeling and Parameter Setup

In addition to experimental investigations, a finite-element model is established as an auxiliary tool to assist in interpreting the deformation and failure behavior of trigger-equipped CFRP tubes.

The present work focuses mainly on experimental observations, and numerical simulation serves as supplementary support rather than the core research content. Material inputs are derived from the lamina test data presented in Section 2.2.1. Parameters for cohesive interlayer elements and the numerical model are summarized in Table 4. In-depth parametric investigations based on this numerical model will be reported in a separate future publication.

The constitutive model, intralaminar failure criterion, cohesive-element-based interlaminar modeling approach and stiffness-degradation scheme adopted in this work follow the modeling framework reported in our previously published work [66]. Specifically, a linear elastic constitutive model is used to characterize the orthotropic mechanical response of each composite lamina. The LaRC05 criterion is employed to predict the onset of intralaminar damage, covering matrix cracking, fiber kinking, fiber splitting and fiber tensile failure. Zero-thickness cohesive elements are embedded between adjacent plies to simulate inter-ply delamination. The quadratic nominal-stress (QUADE) criterion is adopted for interlaminar damage initiation, and an energy-based damage evolution law is utilized to govern the stiffness degradation of cohesive elements. Upon the initiation of intralaminar damage, an exponential progressive stiffness-degradation scheme is implemented for material stiffness reduction. Full theoretical derivations and detailed implementation procedures for the above-mentioned models can be found in Ref. [66] and are not reproduced herein.

The geometric dimensions of the finite-element model are consistent with those of physical specimens. The upper and lower loading platens are modeled as rigid bodies, and general contact is defined for the entire model with a friction coefficient of 0.2. The composite tube is discretized by eight-node linear reduced-integration solid elements (C3D8R). The mesh in the thickness direction is divided strictly according to the actual ply stacking sequence, with one C3D8R element assigned for each composite ply. The in-plane element size is set to 5 mm × 6 mm. Zero-thickness COH3D8 cohesive elements are embedded between adjacent plies to simulate inter-ply delamination. Hourglass control is activated for C3D8R elements to suppress non-physical hourglass deformation under large crushing deformation. A linear displacement-controlled loading scheme is adopted to reproduce quasi-static axial compression conditions. The Abaqus/Explicit solver (Version 2022) (Dassault Systèmes Simulia Corp., Johnston, RI, USA) is employed with reasonable mass scaling to improve computational efficiency. The overall finite-element assembly is displayed in Figure 5.

## 3. Results and Discussion

This section investigates chamfer triggers as a baseline to reveal how the end-defect characteristic size governs crushing initiation behavior.

### 3.1. The Performance of Chamfer Trigger

This group serves as the baseline reference for the subsequent grooved-trigger specimens. Five configurations are investigated in this stage: flat-end, inner chamfer, outer chamfer, double chamfer and fillet chamfer. Their crushing deformation, load–displacement characteristics and crashworthiness performance are analyzed in the following sections.

#### 3.1.1. Load–Displacement Response Characteristics

The load–displacement curves of chamfer specimens are plotted in Figure 6. All configurations in this stage show typical single-peak responses. The flat-end tube generates a prominent initial peak load. Various chamfer triggers can reduce the initial peak to a certain extent by modifying the end geometry. Among the chamfer designs, the double chamfer and fillet chamfer configurations achieve more obvious peak-suppression effects than single-sided chamfers.

For representative extruded chamfer specimens, the numerically predicted load–displacement curve from the finite-element model (Section 2.4) is also imposed in Figure 6. The simulated and experimental load–displacement curves are in good agreement for all chamfer configurations. Due to space limitations, only the results of the representative extruded chamfer specimen are presented herein.

#### 3.1.2. Crashworthiness Indices Evaluation

Key crashworthiness indices (SEA, PCF and CFE) of chamfered specimens are summarized in Table 5 and Figure 7. Since only two repeated specimens were available for each configuration, the results in Table 5 are presented as mean ± relative maximum deviation (%) to quantify data scatter. The relative maximum deviation is defined as the ratio of the maximum absolute deviation of repeated test results to the mean value, expressed as a percentage.

Compared with the flat-end configuration, chamfer modifications effectively reduce the peak crushing force to varying degrees. For internal chamfer and double chamfer specimens, the peak crushing force decreases monotonically with increasing chamfer angle. For extruded external chamfer specimens, PCF decreases first and reaches a minimum value of 41,875.2 N near 45°, followed by a rebound at larger angles. Even so, the peak load of the 75° extruded chamfer specimen (46,155.5 N) is still markedly lower than that of the 15° extruded chamfer (60,736.1 N).

Among all chamfer schemes, double chamfer specimens exhibit competitive comprehensive performance. The Double-30° case achieves the highest SEA value of 77.83 kJ/kg, together with a favorable CFE of 73.4%. For the extruded chamfer series, extruded-30° yields the maximum SEA of 80.65 kJ/kg and a CFE of 79.1%, which stands out among single-sided chamfer specimens. By contrast, large-angle chamfer specimens (60°, 75°) experience obvious degradation in both mean crushing force and energy-absorption capacity. This is consistent with the earlier observation that severe fragmentation and frequent load drops occur for large-angle chamfer samples during crushing.

Fillet chamfer specimens deliver intermediate performance between small-angle and large-angle chamfers. The double-circle CFE reference obtains the highest EA of 2102.1 J and a relatively excellent SEA of 80.85 kJ/kg, while its PCF remains at a high level of 59,070.5 N.

It can be noted that the flat configuration achieves simultaneous low PCF, high mean crushing force and high CFE. Even for the optimal chamfer cases, the improvement in crashworthiness remains limited. As discussed in the previous section, all end-contour-modified specimens undergo single-stage unimodal crushing. Without an independent pre-crushing zone, it is difficult to further decouple the contradiction between peak-load suppression and stable energy-absorption performance. This further motivates the investigation of grooved trigger structures in the subsequent section.

#### 3.1.3. Deformation, Failure Modes and Post-Crushing Morphologies

The digital image correlation (DIC) method was employed to obtain the surface strain fields during crushing. Representative strain contours of test specimens with different chamfers were compared at displacements of 1 mm, 2 mm, 5 mm and 10 mm, as shown in Figure 8. To intuitively reveal the distribution and evolution of high-strain regions in the specimen during loading, a visual recognition method was developed to extract high-strain points frame by frame from DIC images retrieved directly by frame index. An HSV-color-space-based image processing workflow was established to automatically perform contour calibration, valid region segmentation and frame-by-frame detection of the maximum strain point, capturing the time-varying evolution of its strain magnitude and spatial position. The curve of loading time versus the Y-axis coordinate of high-strain points was plotted, as shown in the graph in Figure 8.

For flat-ended tubes, DIC results indicate that discrete and randomly distributed high-strain regions appear on the tube wall during compression, and no fixed deformation band or stable failure path is formed. This irregular strain evolution is consistent with the load–displacement curves in the previous section and explains the higher initial peak load and unstable crushing response of plain-ended CFRP tubes.

For chamfer trigger tubes, DIC observations show that high strain is always concentrated at the loaded end of the tube, without staged strain migration or independent pre-deformation. This single-stage strain characteristic fundamentally results in the single-peak feature of the load–displacement curves. Although the chamfer configuration reduces the randomness of tube failure, it fails to form a staged crushing mechanism and thus exhibits limited improvement in crushing stability.

These DIC observations reveal how trigger geometry modulates strain localization. They offer a process-level explanation for the load–displacement behavior in Section 3.1.1, and also foreshadow the distinct frond-and-fragment morphologies presented in the following section.

Figure 9 presents the top-view crushing morphologies of CFRP tubes with different chamfer configurations and angles. As reported in the existing literature [13,21], triggered CFRP tubes under axial quasi-static compression exhibit two typical frond modes: inward curling towards the inner cavity and outward curling away from the tube walls. Distinct differences in frond geometry, fragmentation degree and damage distribution can be observed in specimens with varied chamfer types and angles.

For internal chamfer specimens (15–75°), fronds mainly curl inward. Specimens with small angles (15°, 30°) show wide and relatively intact fronds with limited brittle fragmentation. As the angle increases to 45–75°, tip-induced damage becomes severe, fiber fracture increases and fronds split into narrow segments.

Both inward and outward fronds can be seen in double chamfer specimens. Well-developed fronds are found at 15° and 30°, whereas severe fiber fragmentation and smaller frond segments occur above 45°. The fillet chamfer reference specimens show intermediate fragmentation between low-angle and high-angle chamfer samples.

These morphological observations provide a physical interpretation of the foregoing load–displacement responses and crashworthiness indices. Meanwhile, they demonstrate that the chamfer trigger can only achieve single-stage progressive crushing. An independent pre-crushing zone cannot be formed ahead of the main crushing region, which limits further performance improvement.

The simulated overall deformation pattern is presented in Figure 9. It can be clearly observed that the traditional continuum-based damage model cannot reproduce the highly random brittle fragmentation and fragment detachment commonly seen in physical experiments, nor can it reproduce the typical petalling opening morphology of triggered specimens. Such mesoscale failure features can be reproduced by means of buckling theory, fracture modeling strategies and other approaches. This morphological comparison intuitively reflects the inherent limitations of the traditional finite-element method in the crushing simulation of composite materials, further highlighting the irreplaceability of experimental characteristics, which serve as the core basis for mechanism analysis in this work.

### 3.2. The Performance of Advanced Trigger Configurations Derived from End-Weakening

As demonstrated in Section 3.1, conventional chamfer-based end triggers only produce single-stage unimodal crushing behavior. The peak-load suppression and stable energy-absorption performance cannot be fully decoupled, owing to the absence of an independent pre-crushing zone before the main crushing region. To address this limitation, two families of advanced trigger geometries are investigated in this section: tulip/crown end-weakening configurations and notch triggers. The load–displacement responses, crashworthiness performance, strain evolution and post-crushing damage morphologies are analyzed to clarify whether segmented bi-modal crushing can be realized by these improved trigger designs.

#### 3.2.1. Load–Displacement Response Characteristics

Figure 10 presents the load–displacement curves of tulip, crown and notch specimens with varying defect quantities. All these trigger configurations adopt purely end-weakening modifications, in which geometric cuts are only machined on the loading terminus while the tube sidewall remains intact. Distinct crushing responses are observed among the three families of specimens even under comparable overall test conditions.

For the tulip and crown series with moderate defect numbers (tulip3–tulip6, crown3–crown6), a representative two-phase crushing behavior can be clearly identified. After the initial loading stage, an early pre-crushing peak occurs, followed by an obvious load valley induced by the bending deformation of cantilever petals at the tube end. Subsequently, the load gradually recovers and transitions into a relatively stable crushing plateau corresponding to the progressive main crushing of the tube wall. As the number of cut-formed cantilever petals increases, the magnitude of the valley load decreases, accompanied by more pronounced high-frequency load oscillations throughout the crushing plateau. These oscillations are associated with continuous deformation disturbances of individual end petals. When the defect number rises to eight (tulip8, crown8), the equivalent end-weakening exceeds the critical upper threshold. The deformation process of end petals and the main crushing of the tube wall largely overlap in the time domain. The bimodal two-phase signature disappears, producing an apparent single-peak load response. Notably, the peak positions of tulip8 and crown8 shift to approximately 10 mm displacement, rather than occurring near the origin as observed for the baseline chamfer specimen.

Different from tulip and crown configurations, notch specimens only introduce shallow local indentations at the tube end without generating separated free-standing cantilever petals, and the circumferential rim of the tube remains structurally continuous. For the whole notch series ranging from notch3 to notch10, no well-defined two-phase pre-crushing valley can be observed on load–displacement curves. All notch specimens exhibit intrinsic single-peak characteristics similar to the chamfer reference case, where the major load peak appears at a small displacement close to zero, regardless of the notch quantity. Even for notch8, which shares the same defect count with tulip8 and crown8, the load–displacement behavior remains fundamentally different. This direct comparison strongly demonstrates that the crushing transition is governed by the equivalent severity of end defects, instead of merely the number of cuts or indentations. It should be emphasized that equivalent end-weakening serves as a primary metric, and geometric configuration also exerts an independent influence on crushing responses.

Two physically distinct mechanisms can produce an apparent single-peak load profile in these end-modified tubes. On one hand, insufficient end-weakening (e.g., all notch specimens) fails to activate an independent pre-crushing phase; deformation directly propagates into the tube wall, yielding an intrinsic single-peak response. On the other hand, excessive end-weakening beyond the upper critical threshold (e.g., tulip8, crown8) leads to temporal overlap between end-petal deformation and main crushing, which also eliminates the bimodal feature and produces an apparent single-peak curve with a delayed peak displacement.

It can also be observed that within the valid working window of equivalent end-weakening, tulip triggers generally maintain a higher plateau load level compared with crown counterparts under identical defect numbers. Nevertheless, even for specimens showing favorable bimodal responses, pre-crushing and main crushing are only separated in the time domain. Damage initiation is strictly confined to the loading end face for all the above configurations, and complete decoupling of pre-crushing and main crushing cannot be achieved spatially.

#### 3.2.2. Crashworthiness Indices Evaluation

Key crashworthiness indices of tulip-, crown- and notch-triggered specimens are quantitatively evaluated in Table 6, including total energy absorption (EA), peak crushing force (PCF), average crushing load (Pm), crushing load efficiency (CFE) and specific energy absorption (SEA). The results in Table 6 are presented as mean ± relative maximum deviation (%) to quantify data scatter. It should be noted that for bimodal load–displacement curves, PCF is defined as the maximum of the two peaks, rather than the first local minor peak originating from end triggering. The parametric variation trends are displayed in Figure 11, which are further supplemented and explained by the load–displacement characteristics summarized in Section 3.2.1.

For tulip and crown configurations, the overall crashworthiness performance varies regularly with the change in end cut geometry. Specimens with moderate cutting dimensions exhibit preferable comprehensive performance, which corresponds to the stable two-stage load response observed in the load–displacement curves. Within this geometric range, both series maintain a high level of crushing load efficiency. The CFE of the tulip series decreases slightly from 79.7% (tulip3) to 76.9% (tulip6), while the crown series decreases from 78.6% (crown3) to 60.4% (crown5). The gradual reduction in peak crushing force indicates that properly arranged end petals can effectively alleviate the initial load overshoot. However, when the number of end cuts increases to eight, the comprehensive performance deteriorates significantly. Although the peak load does not rise sharply, the CFE values decline to 73.3% and 57.6% for tulip8 and crown8, respectively. This performance degradation is consistent with the disappearance of the two-stage load feature, where the overlapping deformation procedure weakens the cooperative relationship between initial load response and subsequent stable crushing behavior.

Consistent with the load–displacement analysis in Section 3.2.1, the present index results confirm that the crushing behavior and final crashworthiness arise from the coupled effects of geometric configuration and the equivalent severity of end-region modification. Two different physical pathways lead to single-peak load profiles and poor crushing efficiency. Notch specimens, characterized by a continuous circumferential rim and no free-standing cantilever petals, cannot trigger an independent pre-crushing phase and deliver intrinsic single-peak responses. By contrast, tulip8 and crown8 lose bimodal signatures owing to the temporal superposition of end-petal deformation and main-tube crushing, generating apparent single-peak curves with delayed peak displacement.

Even for specimens with stable two-stage crushing responses, pre-crushing deformation only occurs temporally prior to main-tube crushing. Since all deformation activities are concentrated at the tube end without axially independent crushing zones, the deformation coupling between end petals and tube wall cannot be completely eliminated. This inherent structural limitation restricts the further improvement of load coordination and crushing stability, which explains why the crashworthiness performance of purely end-modified configurations cannot be substantially enhanced despite the geometric optimization of end features.

#### 3.2.3. Deformation, Failure Modes and Post-Crushing Morphologies

DIC was adopted to record the time-resolved evolution of the surface strain field for tulip-, crown- and notch-triggered CFRP tubes during quasi-static crushing. Time-sequential strain contours of a representative tulip specimen are given in Figure 12, covering 1 mm, 2 mm, 5 mm and 10 mm after compression initiation. At this early crushing stage, damage originates from the end-machined trigger features, while speckle loss induced by fragment spalling remains limited.

Tulip and crown triggers with a moderate number of cutouts present a typical two-stage strain evolution behavior. High strain firstly concentrates on the end petals and induces petal bending deformation, and then transfers to the upper tube wall to trigger progressive crushing. This strain evolution matches well with the bimodal load–displacement response, in which the first peak corresponds to petal pre-crushing and the second peak corresponds to the primary crushing of the tube wall.

For tulip and crown tubes with eight notches, DIC measurements reveal synchronous deformation of end petals and adjacent tube walls. High-strain regions continuously distribute on the upper tube wall below the loaded end, and no discrete high-strain spots are observed. This strain feature explains the single-peak response and the delayed peak displacement of approximately 10 mm. Excessive end-weakening reduces the stiffness difference between end petals and the tube wall, eliminates the independent pre-crushing stage, and eventually leads to synchronous and controllable crushing behavior.

The macroscopic top-view post-crushing morphologies of this group are shown in Figure 13. All specimens experience end-initiated progressive crushing and exhibit typical frond-dominated failure modes. For tulip specimens with moderate cut quantities (tulip3, tulip6), relatively intact and broad fronds are observed with moderate fiber fragmentation. Each pre-cut segment opens and bends outward, forming uniformly distributed petal-like fronds. Fiber and matrix damage mainly occurs at petal roots, and large detached fragments are seldom seen. As the number of end cuts increases to eight, individual petals become narrower, fronds are divided into smaller segments, and fine fragmented debris increases significantly.

Notch specimens show distinct crushing features. Without pre-fabricated cantilever segments, the fronds are irregular with variable sizes. Increasing the notch quantity fails to divide the tube end into independent deformable units, and frond tearing and random fragmentation become severe. Severe local splitting and abundant fine debris are observed for notch8, notch9 and notch10.

Time-resolved DIC strain results combined with post-crushing morphologies give physical interpretations for the above-mentioned experiments. The two types of single-peak responses originate from different mechanisms: notch tubes show intrinsic single-peak behavior due to the absence of independent pre-crushing units, while the single-peak curves of tulip8 and crown8 result from synchronous multi-site damage superposition. It is verified that crushing behavior and crashworthiness are governed by the equivalent modification degree of the tube end rather than the sheer number of cuts or notches. Pure end-modified triggers (petal-type or shallow-notch-type) can only adjust the damage sequence within the tube end and cannot produce an axially isolated pre-crushing zone spatially separated from the main crushing region. Accordingly, composite trigger schemes combining end-weakening with coupled structural features represent a promising direction for further research.

## 4. Conclusions

To address the limitation that existing investigations into end-triggered CFRP energy-absorbing tubes largely focus on individual trigger configurations and lack systematic comparative research under a unified experimental benchmark, this study performs quasi-static axial crushing tests on thin-walled CFRP tubes featuring four typical end-weakening trigger geometries—chamfer, tulip, crown, and notch—under identical material systems, geometric dimensions, and loading conditions. With the assistance of DIC full-field deformation measurement and visual recognition post-processing, the dynamic evolution of strain fields and high-strain regions throughout the entire crushing process was accurately captured in this study. Combined with post-crushing morphologies, the dominant mechanism of the equivalent end-weakening effect on the crushing behavior and crashworthiness of CFRP tubes was analyzed. The main conclusions are drawn as follows:

1. Chamfer modification can effectively reduce the initial peak crushing load of CFRP tubes. The evolution of peak load with chamfer angle differs markedly for various chamfer configurations. A moderate chamfer angle can optimize energy absorption performance and realize both peak suppression and improved energy absorption. Nevertheless, excessively large chamfer angles aggravate the end-weakening effect and lead to a reduction in mean crushing force and total energy absorption. Improvements in crashworthiness relying solely on end contour modification have inherent limitations.
2. The configuration of trigger structures significantly affects the crushing response. Within a reasonable parameter range, tulip and crown triggers with discrete cantilever petals achieve a stable two-stage bimodal crushing response. Excessive cutting leads to an over-limit end-weakening degree, transforming the crushing response into a single-peak mode. By contrast, notch triggers always exhibit an inherent unimodal crushing characteristic (even when the material removal area of notch specimens is equivalent to that of Tulip4). Notch6, one of the notch specimens, delivers a total energy absorption of up to 2186.2 J, the highest among all specimens. However, this category of structures generally suffers from a high peak crushing load and low crushing force efficiency, showing an obvious performance trade-off. These observations demonstrate that geometric configuration and equivalent end-weakening exert a bidirectional coupled regulatory effect on crushing behavior.
3. The crushing-mode transition and crashworthiness of CFRP tubes are jointly governed by geometric configuration and the equivalent end-weakening degree. Insufficient end-weakening induces inherent single-peak failure, while excessive end-weakening causes synchronous superposition of multi-site damage and further forms an apparent single-peak response. End material removal and stiffness reduction caused by machining parameters such as chamfer size and cutout quantity serve as the core factors governing the strain evolution and crushing characteristics of modified tubes. Future studies will further distinguish the independent effects of trigger geometric features and end-weakening degrees to reveal the intrinsic mechanism of crushing mode transition.
4. Existing end-weakening triggers possess simple configurations and convenient machinability. However, pure end modification restricts deformation and damage to the tube end region and cannot realize axial multi-stage crushing, which limits the further improvement of crashworthiness. Composite trigger designs combining end-weakening with coupled structural features provide an effective approach to optimize the crushing stability and energy-absorbing performance of CFRP tubes in future research.
5. The finite-element model established in this study can accurately predict the macroscopic crushing mechanical responses of end-modified CFRP tubes, but the conventional continuum-based method limits its capability to reproduce experimental petalling morphology and random brittle fragmentation. In future work, buckling theory and fracture modeling strategies will be employed to improve the numerical prediction accuracy of meso-scale failure behaviors.

## Figures and Tables

**Figure 1:**
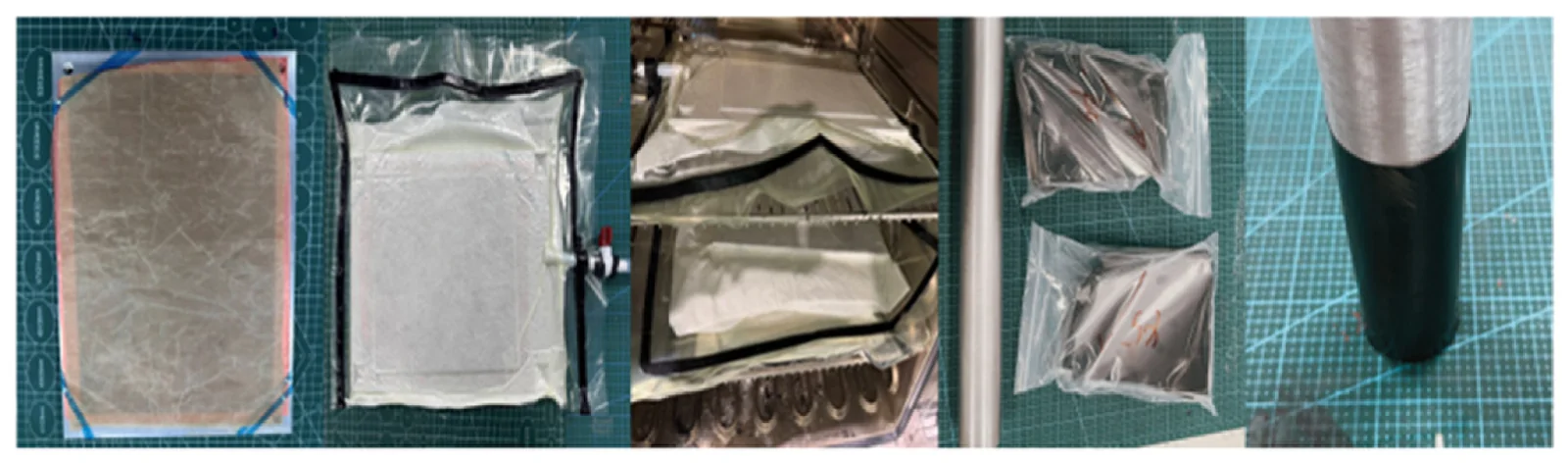
The test specimen preparation process.

**Figure 2:**
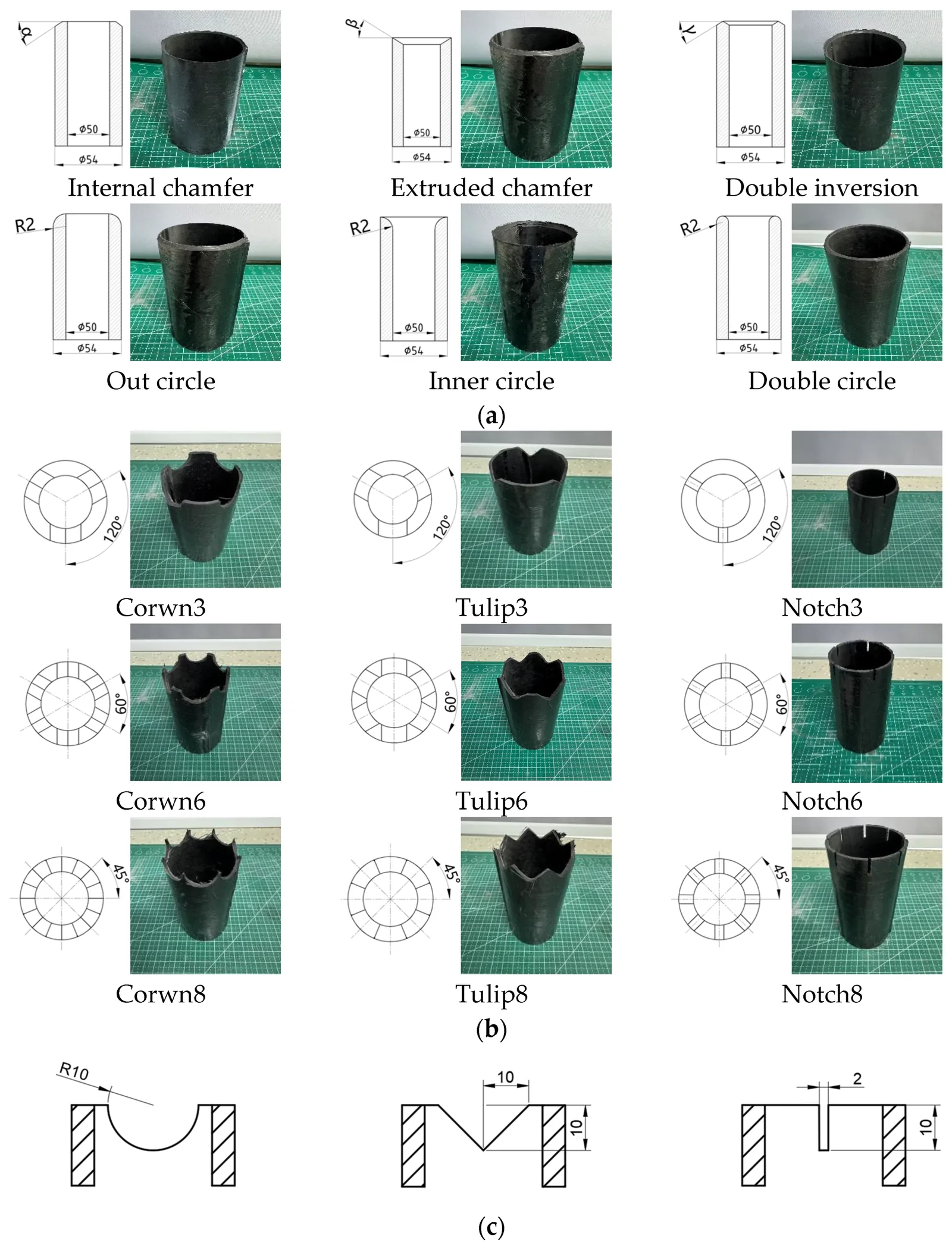
Test specimens with geometric dimensions and triggering design: (**a**) the chamfer trigger; (**b**) tulip/crown and notch triggers; (**c**) induced defect geometry.

**Figure 3:**
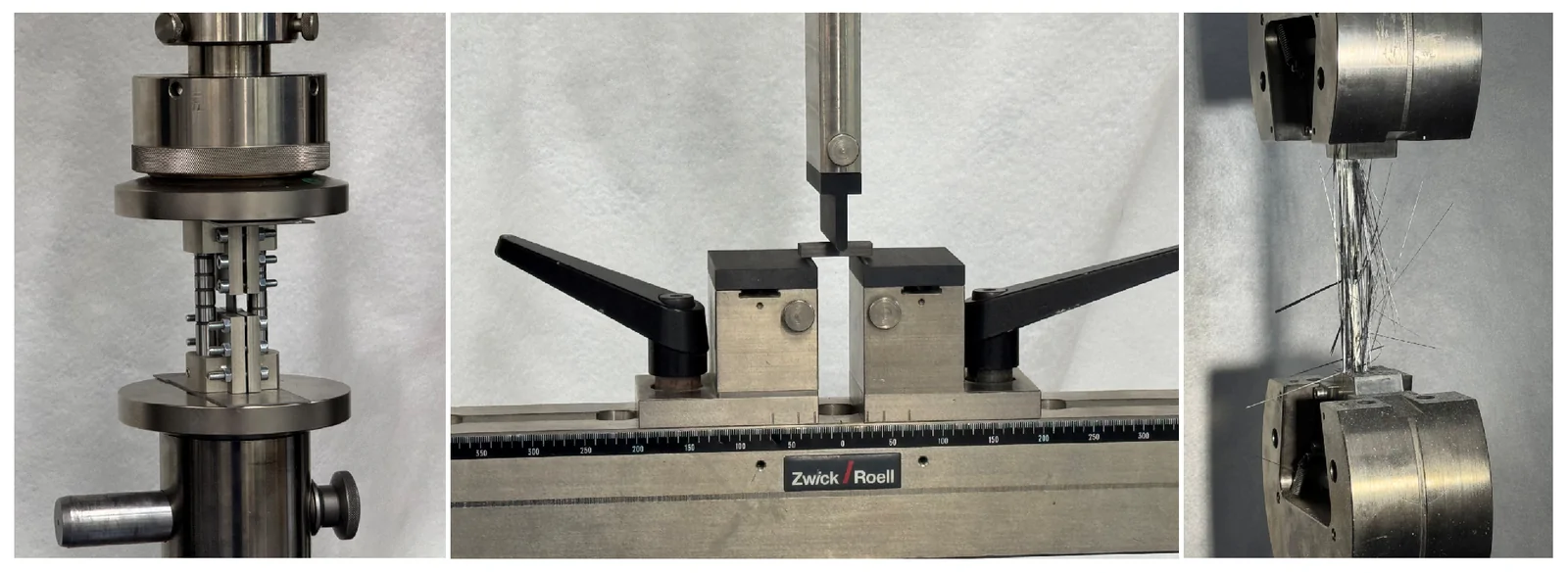
Single-layer mechanical property test.

**Figure 4:**
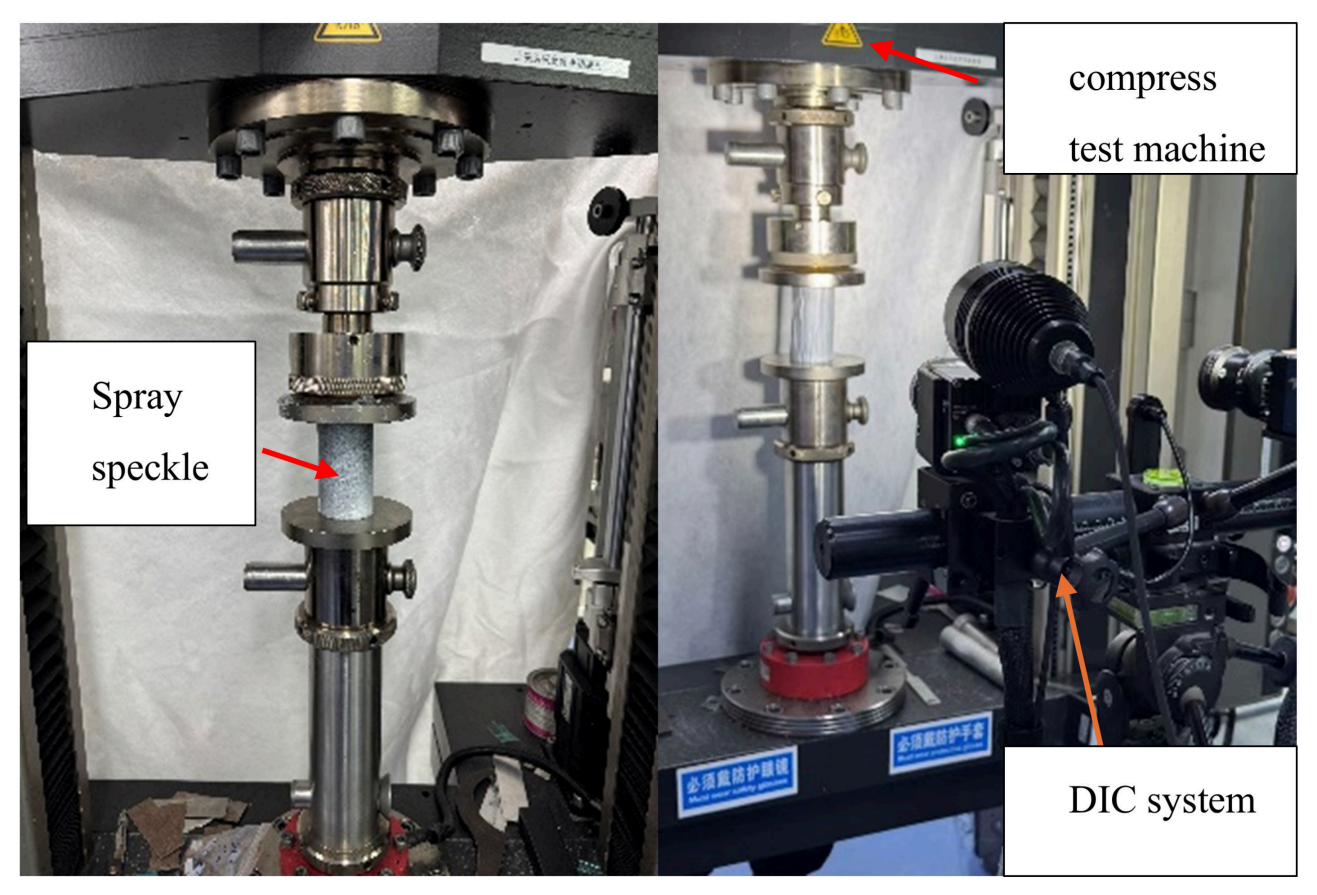
Compression test apparatus integrated with the DIC system.

**Figure 5:**
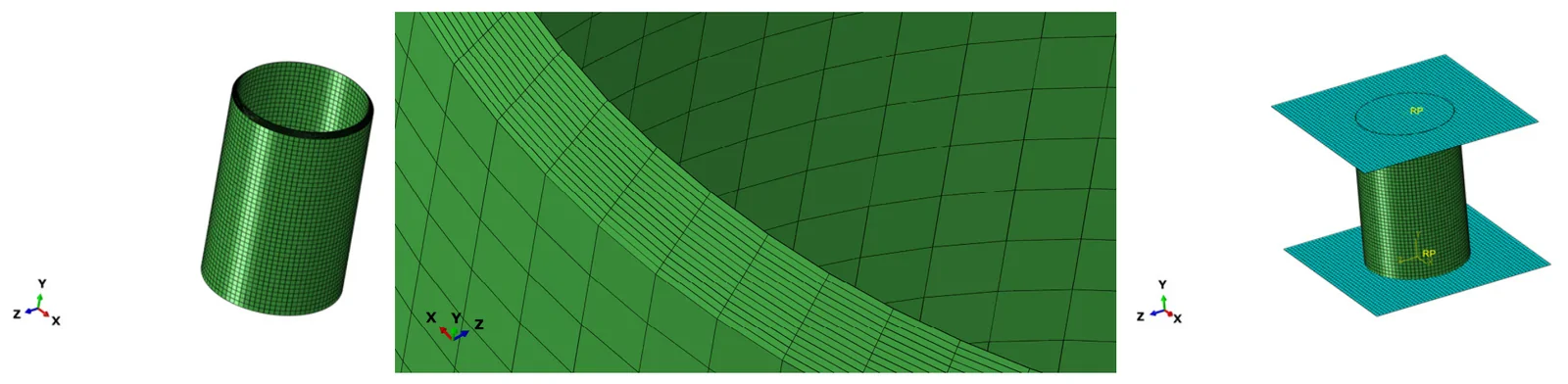
Finite-element model of the tube under quasi-static axial compression.

**Figure 6:**
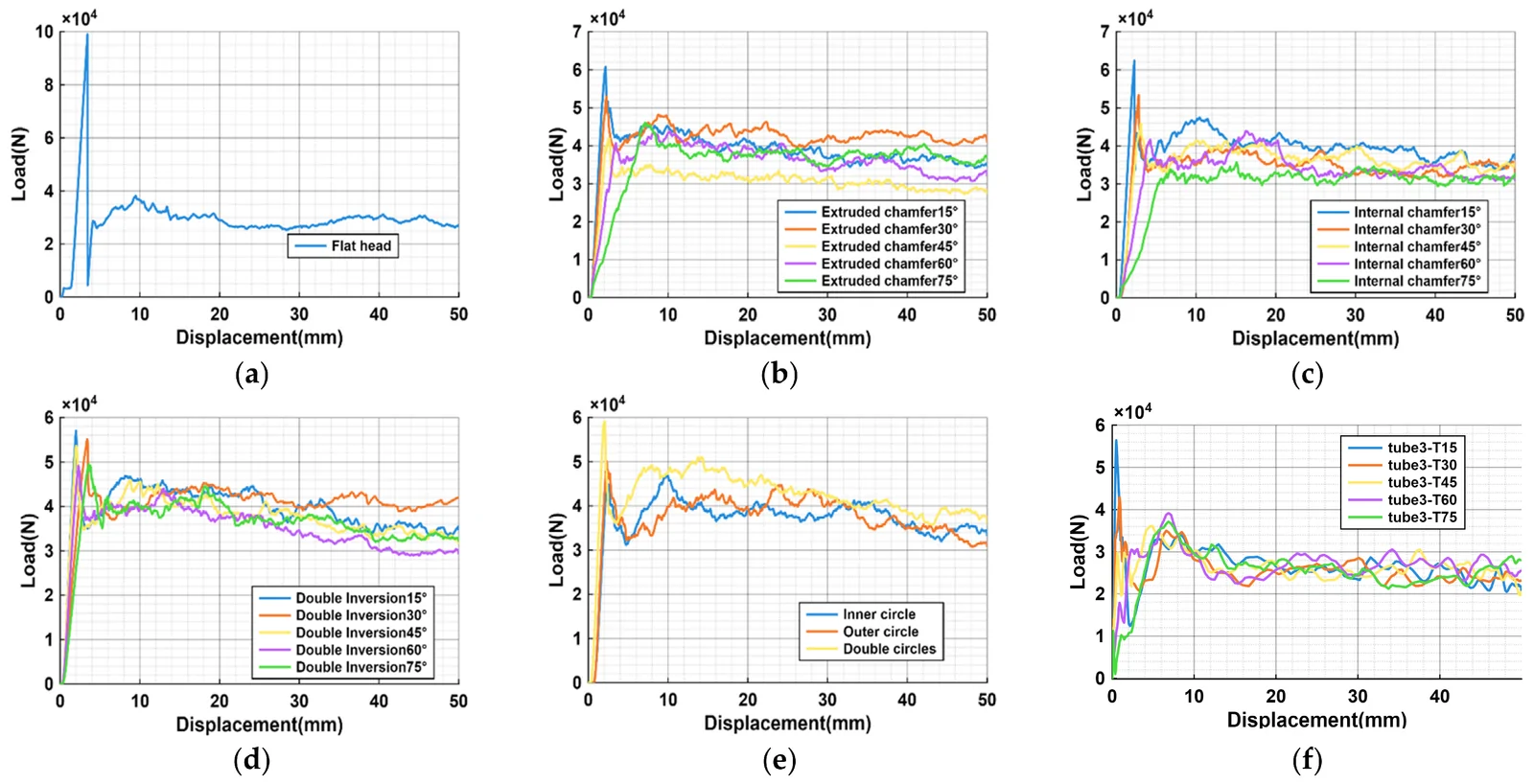
Load–displacement curves of chamfer specimens: (**a**) flat head; (**b**) extruded chamfer; (**c**) internal chamfer; (**d**) double chamfer; (**e**) filler chamfer; (**f**) the simulated result of extruded specimens.

**Figure 7:**
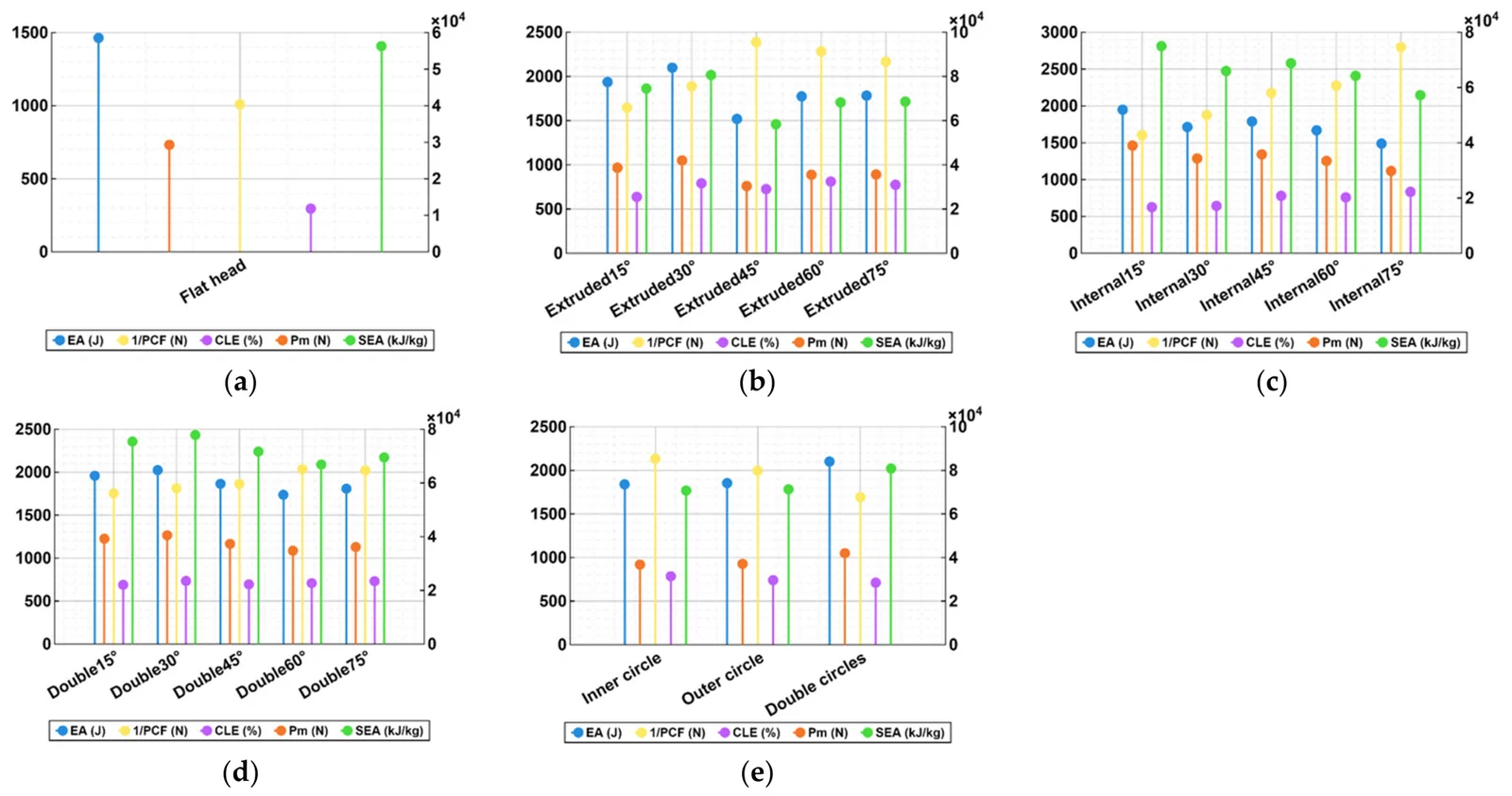
Key crashworthiness indices for each specimen in chamfer: (**a**) flat end; (**b**) extruded chamfer; (**c**) internal chamfer; (**d**) double chamfer; (**e**) fillet chamfer.

**Figure 8:**
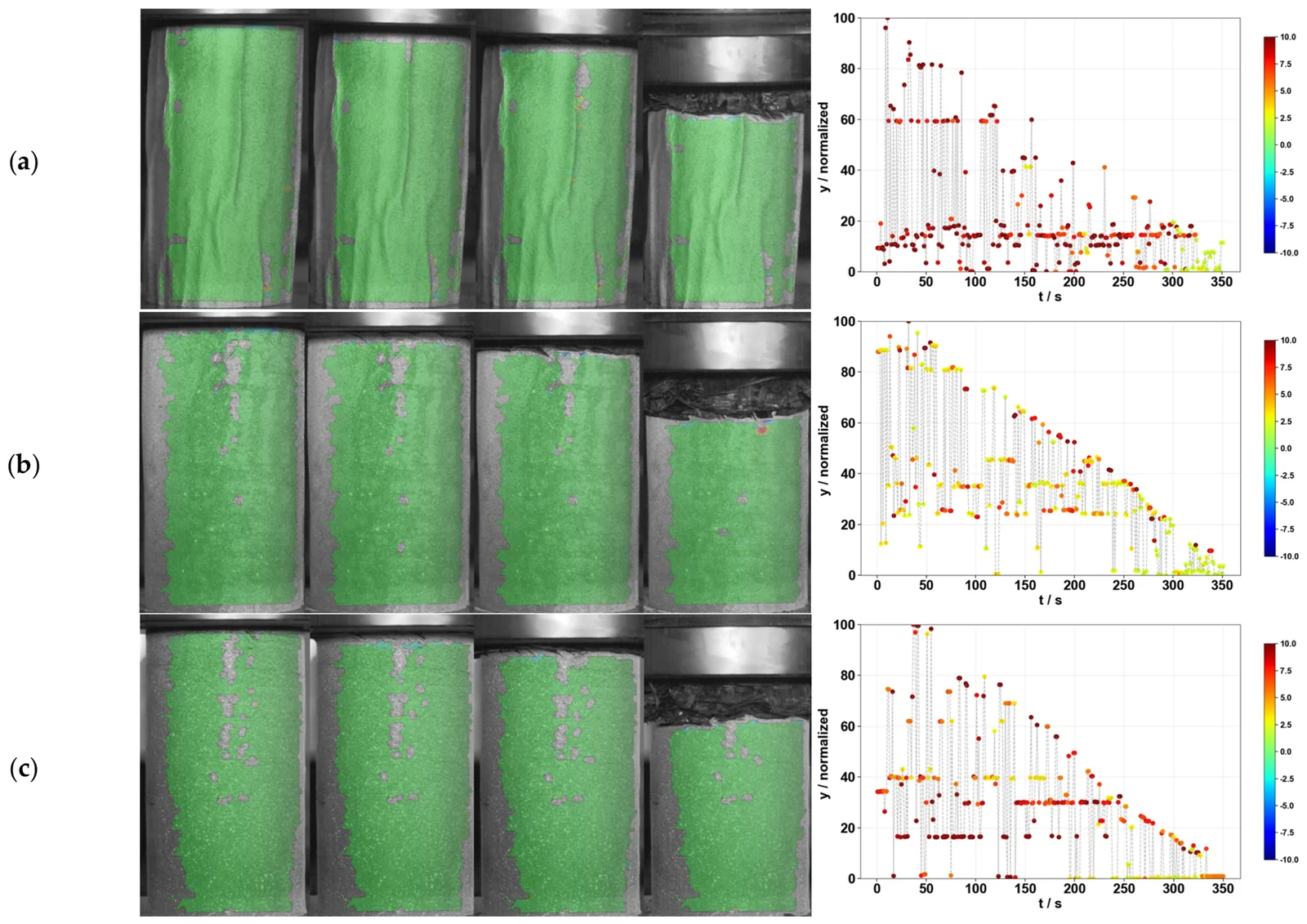
The strain field of a typical specimen via a Digital Image Correlation (DIC) system: (**a**) Flat end; (**b**) extruded 30; (**c**) double circle. The green area represents the valid calculation region obtained by the DIC analysis.

**Figure 9:**
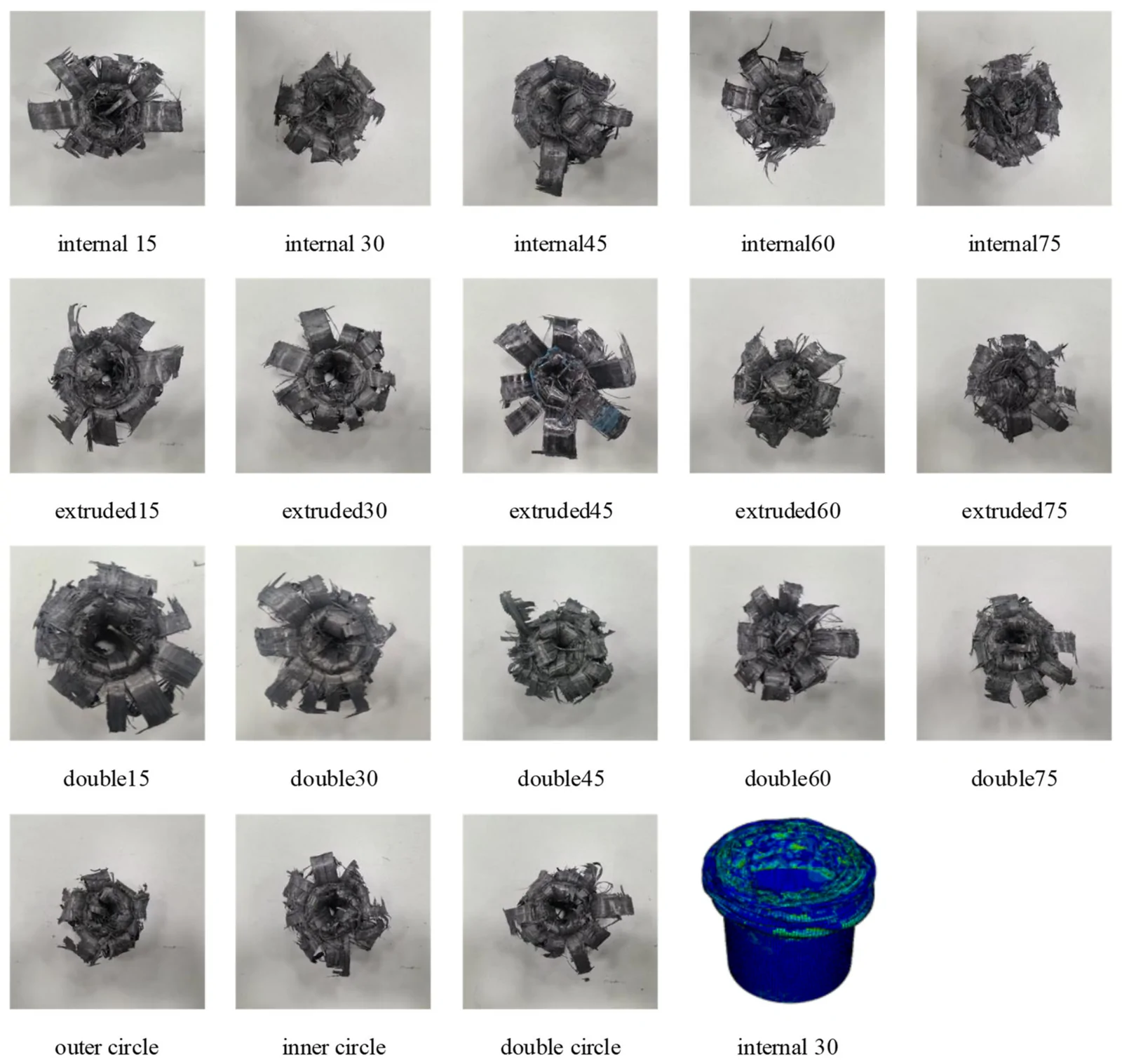
Macroscopic post-crushing morphologies. The color contour represents the strain distribution: blue indicates low stress, while green denote high stress regions.

**Figure 10:**
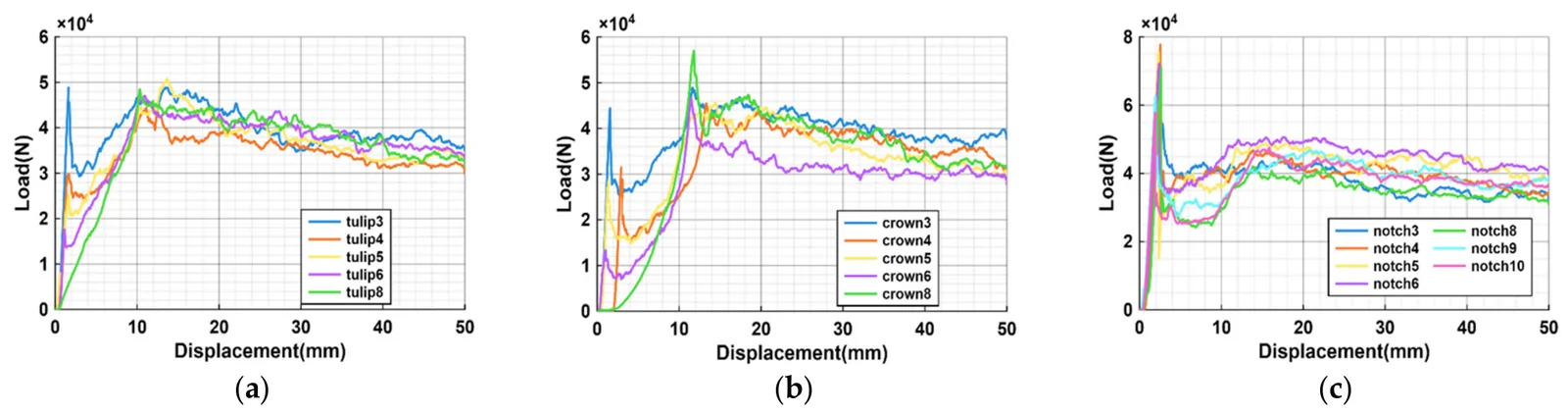
Load–displacement response: (**a**) tulip; (**b**) crown; (**c**) notch.

**Figure 11:**
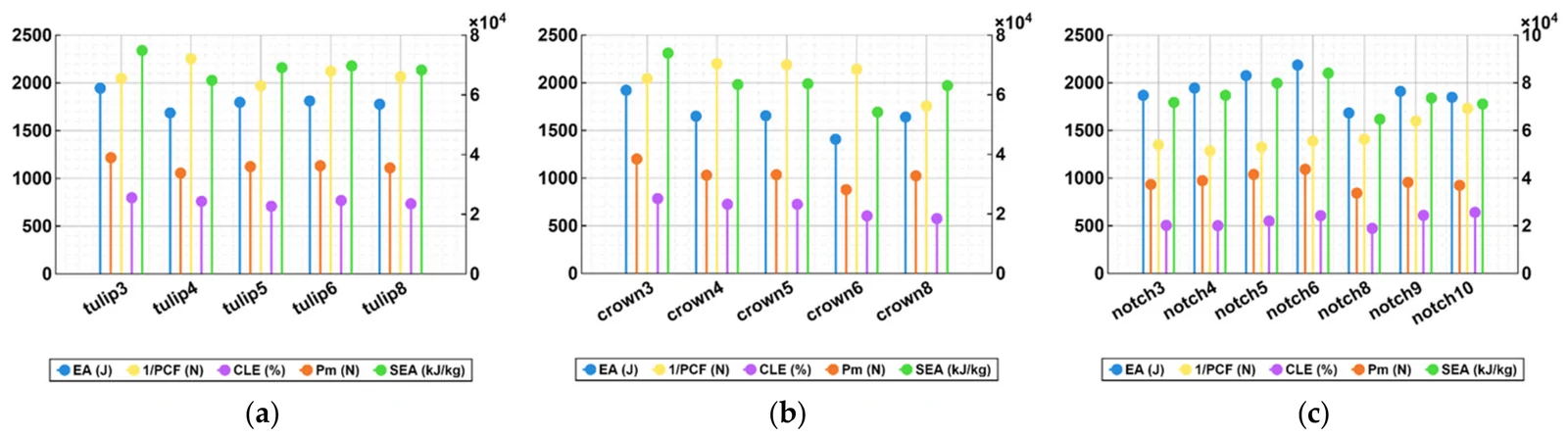
Comparison of the energy absorption characteristics for different trigger specimens: (**a**) tulip; (**b**) crown; (**c**) notch.

**Figure 12:**
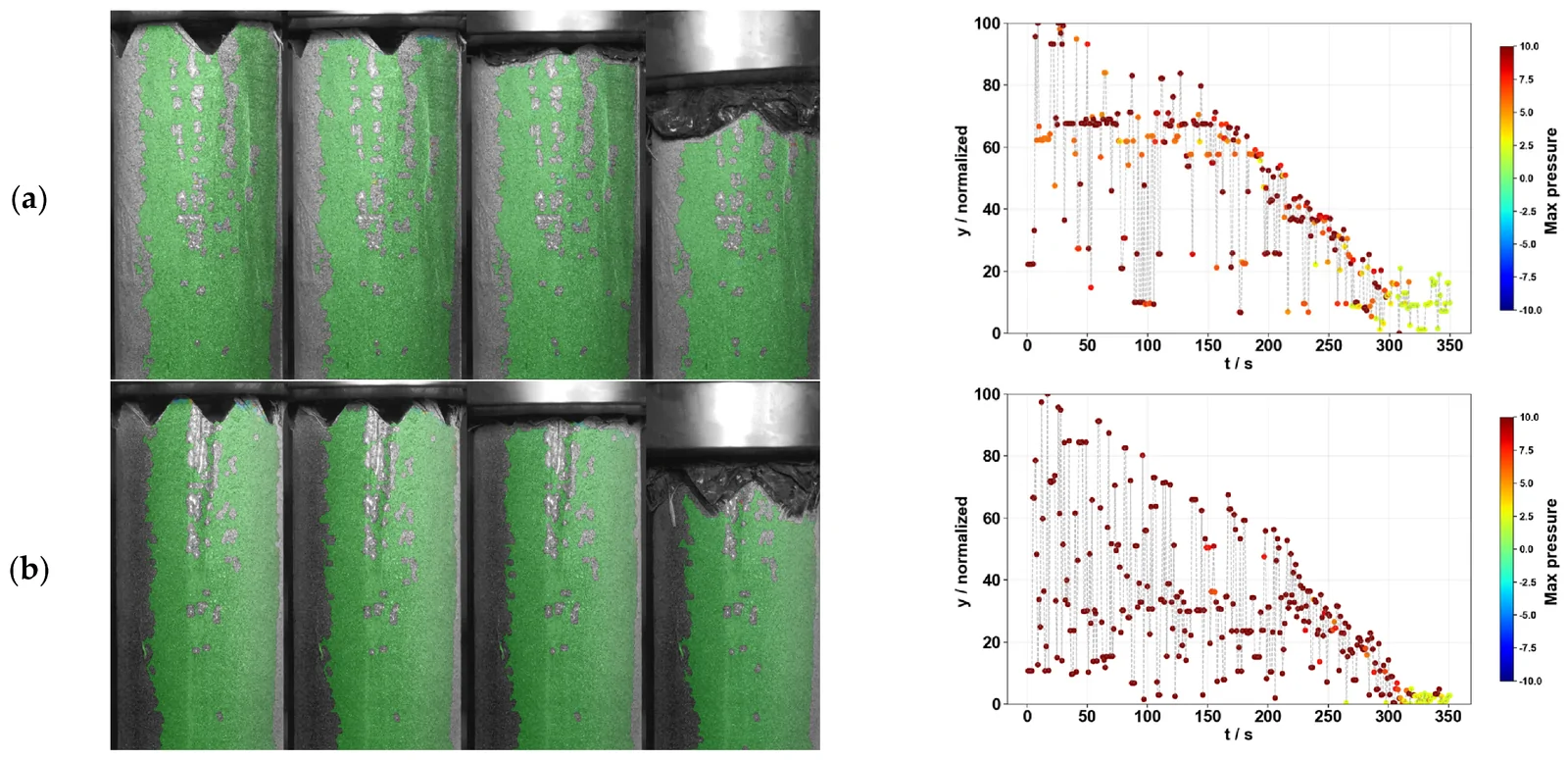

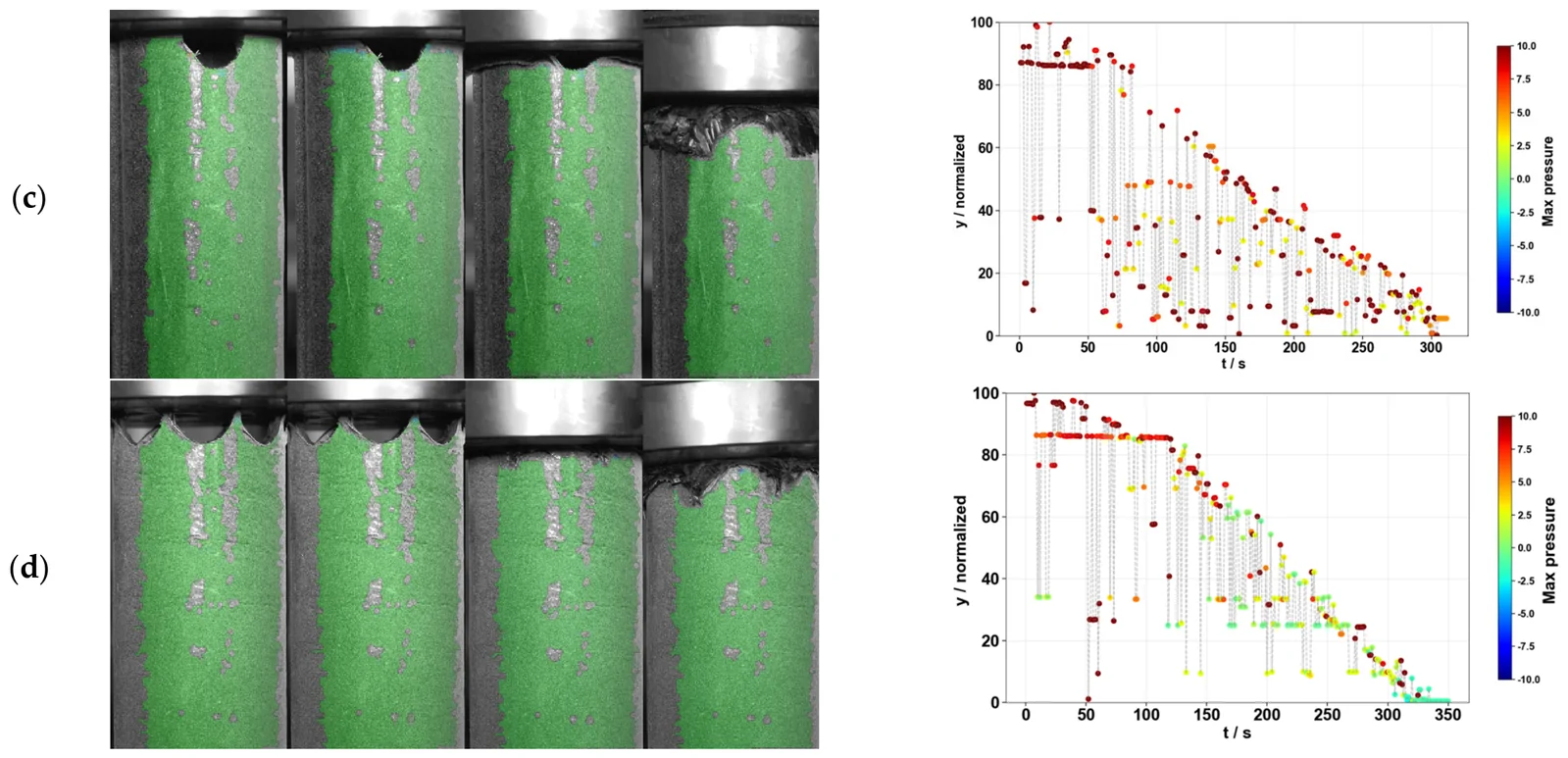
The strain field of partial test specimens via a Digital Image Correlation (DIC) system: (**a**) tulip5; (**b**) tulip8; (**c**) crown 3; (**d**) crown8. The green area represents the valid calculation region obtained by the DIC analysis.

**Figure 13:**
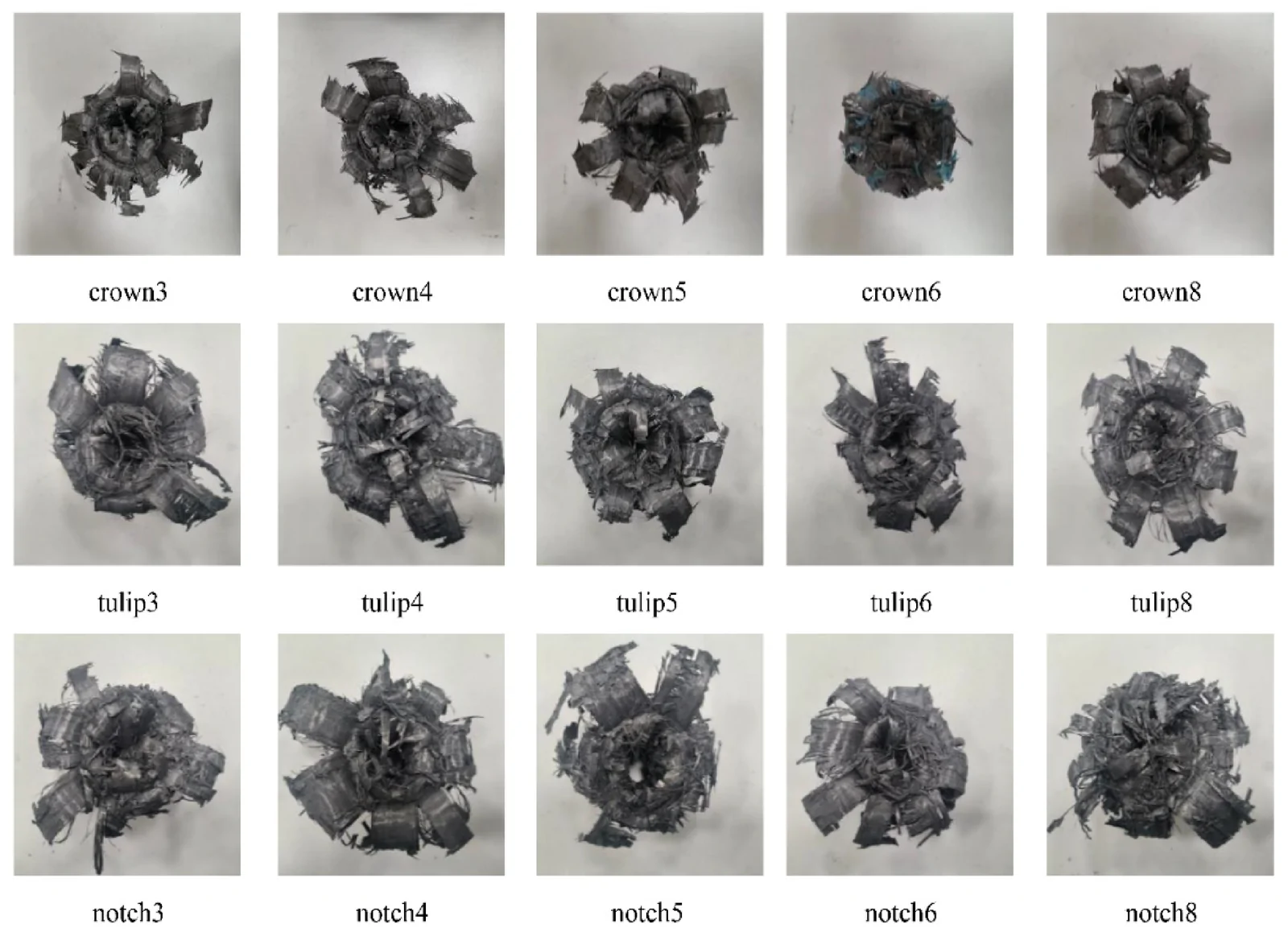
Macroscopic top-view post-crushing morphologies of partial test specimens.

**Table 1:** Test specimen number and description.

Number	Feature	Explanation	Total
Flat	No chamfer	/	2
Extruded 15/30/45/60/75	Extruded chamfer	The angles of the chamfer are respectively: 15°/30°/45°/60°/75°	10
Internal 15/30/45/60/75	Internal chamfer		10
Double 15/30/45/60/75	Double chamfer		10
Outer circle	Outer fillet	The fillet radius is 2 mm.	6
Inner circle	Internal fillet		
Double circle	Double fillet		
Tulip 3/4/5/6/8	Tulip trigger	The defect is in the form of a triangle, uniformly divided into 360 degrees. The number of defects is 3/4/5/6/8 respectively.	10
Crown 3/4/5/6/8	Crown trigger	The defect is in the form of semi-circular, uniformly divided into 360 degrees. The number of defects is 3/4/5/6/8 respectively.	10
Notch 3/4/5/6/8/9/10	Notch trigger	The defect is in the shape of a rectangle and is evenly divided into 360 degrees. The number of defects is 3/4/5/6/8/9/10 respectively.	14
Total numbers		36 different types of test specimens	72

**Table 2:** Equivalent end-weakening geometric removal volumes of specimens.

Number of Notches	*Veq* (mm^3^)		
	Tulip	Crown	Notch
3	300	942	120
4	400	1256	160
5	500	1570	200
6	600	1884	240
8	700	2514	320
9	/	/	360
10	/	/	400

**Table 3:** Material properties of the unidirectional composite laminates.

Symbol	Description	Value
*E*_11_/GPa	Young’s modulus along longitudinal direction	139.87
*E*_22_/GPa	Young’s modulus along transverse direction	8.85
*E*_33_/GPa	Young’s modulus in thickness direction	8.85 *
*ν*_12_	Poisson’s ratio	0.27
*ν*_13_	Poisson’s ratio	0.27
*ν*_23_	Poisson’s ratio	0.27 *
*X_T_*/MPa	Longitudinal tensile strength	2155.35
*X_C_*/MPa	Longitudinal compressive strength	463.08
*Y_T_*/MPa	Transverse tensile strength	45.89
*Y_C_*/MPa	Transverse compressive strength	123.74
*S_L_*/MPa	In-plane shear strength	53.39
*S_T_*/MPa	Transverse shear strength	54.95 *

Note: * indicates the value from calculating, and the rest are values from tests and literature.

**Table 4:** The parameters of the simulation model.

Symbol	Description	Value
$\alpha_{0}$	Fracture plane angle for pure compression	53°
$\varphi_{c}$	Misalignment angle at failure for pure compression	2.0628 *
$\eta_{L}$	Longitudinal shear friction coefficient	0.1595 *
$\eta_{T}$	Transverse shear friction coefficient	0.2867 *
$G_{IC}$/(J/m^2^)	Interlayer fracture toughness of Mode-I	220
$G_{IIC}$/(J/m^2^)	Interlayer fracture toughness of Mode-II	760
X/MPa	Longitudinal strength of interlayer	40
S/MPa	Shear strength of interlayer	80
K/(N/mm^3^)	Penalty stiffness	1.6 × 10^6^
*G*_12_/GPa	In-plane shear modulus	4.8 *
*G*_13_/GPa	Shear modulus in 1–3 plane	4.8 *
*G*_23_/GPa	Shear modulus in 2–3 plane	4.8 *

Note: * indicates the value from calculating, and the rest are values from tests and literature.

**Table 5:** Key crashworthiness indices for chamfered specimens and flat.

Serial Number	EA (J)	Pm (N)	PCF (N)	CFE (%)	SEA (J/kg)	PCF-Sim (N)
Flat	1801.2 ± 37.6%	36,024.6 ± 37.6%	74,988.0 ± 64.1%	56.8 ± 95.9%	69,278.0 ± 37.6%	84,592.7 (0.13)
Double15°	1959.8 ± 0.6%	39,195.6 ± 0.6%	56,965.4 ± 1.4%	68.8 ± 2.1%	75,376.1 ± 0.6%	53,010.9 (−0.07)
Double30°	2023.4 ± 0.0%	40,469.5 ± 0.0%	55,121.6 ± 0.0%	73.4 ± 0.0%	77,826.1 ± 0.0%	53,338.2 (−0.03)
Double45°	1864.5 ± 3.5%	37,289.4 ± 3.5%	53,605.7 ± 1.4%	69.6 ± 4.9%	71,710.4 ± 3.5%	44,715.8 (−0.17)
Double60°	1738.2 ± 5.8%	34,763.8 ± 5.8%	49,108.2 ± 1.2%	70.8 ± 4.6%	66,853.5 ± 5.8%	61,527.9 (0.25)
Double75°	1808.1 ± 6.8%	36,162.4 ± 6.8%	49,477.6 ± 7.0%	73.1 ± 0.3%	69,543.1 ± 6.8%	53,214.3 (0.08)
Internal15°	1949.6 ± 1.2%	38,992.4 ± 1.2%	62,409.3 ± 3.6%	62.5 ± 2.5%	74,985.5 ± 1.2%	48,689.7 (−0.22)
Internal30°	1714.4 ± 5.8%	34,287.4 ± 5.8%	53,327.9 ± 4.9%	64.3 ± 10.7%	65,937.3 ± 5.8%	46,484.7 (−0.13)
Internal45°	1788.3 ± 5.3%	35,765.1 ± 5.3%	45,933.8 ± 0.9%	77.9 ± 4.3%	68,779.0 ± 5.3%	38,236.8 (−0.17)
Internal60°	1669.4 ± 8.7%	33,388.9 ± 8.7%	43,927.1 ± 6.9%	76.0 ± 1.8%	64,209.3 ± 8.7%	39,146.0 (−0.11)
Internal75°	1487.0 ± 12.3%	29,739.3 ± 12.3%	35,733.5 ± 20.0%	83.2 ± 7.8%	57,190.9 ± 12.3%	35,589.2 (0.00)
Extruded15°	1936.7 ± 5.4%	38,733.2 ± 5.4%	60,736.1 ± 7.4%	63.8 ± 12.8%	74,486.8 ± 5.4%	56,417.0 (−0.07)
Extruded30°	2096.8 ± 4.4%	41,935.6 ± 4.4%	53,003.4 ± 7.2%	79.1 ± 2.8%	80,645.4 ± 4.4%	42,978.2 (−0.19)
Extruded45°	1518.1 ± 14.9%	30,362.8 ± 14.9%	41,875.2 ± 8.9%	72.5 ± 6.1%	58,390.1 ± 14.9%	37,441.0 (−0.11)
Extruded60°	1775.1 ± 0.6%	35,502.4 ± 0.6%	43,782.5 ± 1.7%	81.1 ± 1.1%	68,273.8 ± 0.6%	39,195.0 (−0.10)
Extruded75°	1783.3 ± 0.8%	35,666.4 ± 0.8%	46,155.5 ± 1.0%	77.3 ± 0.2%	68,589.2 ± 0.8%	37,165.0 (−0.19)
Inner circle	1840.6 ± 4.0%	36,812.8 ± 4.0%	46,890.4 ± 5.3%	78.5 ± 1.3%	70,793.8 ± 4.0%	38,912.4 (−0.17)
Outer circle	1855.0 ± 1.4%	37,099.5 ± 1.4%	50,079.8 ± 0.5%	74.1 ± 1.9%	71,345.3 ± 1.4%	42,531.8 (−0.15)
Double circles	2102.1 ± 0.5%	42,042.6 ± 0.5%	59,070.5 ± 4.2%	71.2 ± 3.7%	80,851.2 ± 0.5%	49,357.6 (−0.16)

Note: Columns PCF-sim are uniformly expressed in the format of “simulated value (relative error of simulation with respect to experiment)”.

**Table 6:** Key crashworthiness indices of different trigger specimens.

Serial Number	EA (J)	Pm (N)	PCF (N)	CFE (%)	SEA (J/kg)	PCF-Sim (N)
tulip3	1945.9 ± 5.3%	38,918.3 ± 5.3%	48,849.3 ± 4.8%	79.7 ± 0.4%	74,842.9 ± 5.3%	57,906.0 (0.19)
tulip4	1686.7 ± 8.6%	33,733.2 ± 8.6%	44,339.0 ± 6.1%	76.1 ± 2.5%	64,871.6 ± 8.6%	55,437.5 (0.25)
tulip5	1797.3 ± 1.1%	35,946.8 ± 1.1%	50,766.1 ± 4.8%	70.8 ± 3.7%	69,128.4 ± 1.1%	44,819.4 (−0.12)
tulip6	1812.1 ± 1.2%	36,241.1 ± 1.2%	47,107.0 ± 3.1%	76.9 ± 2.0%	69,694.4 ± 1.2%	56,185.4 (0.19)
tulip8	1775.6 ± 6.2%	35,512.1 ± 6.2%	48,440.3 ± 2.8%	73.3 ± 3.5%	68,292.5 ± 6.2%	58,778.2 (0.21)
crown3	1922.3 ± 1.5%	38,446.1 ± 1.5%	48,900.6 ± 9.0%	78.6 ± 7.4%	73,934.8 ± 1.5%	55,970.5 (0.14)
crown4	1649.3 ± 0.4%	32,986.2 ± 0.4%	45,451.0 ± 1.4%	72.6 ± 1.8%	63,435.0 ± 0.4%	47,791.4 (0.05)
crown5	1654.9 ± 3.6%	33,098.1 ± 3.6%	45,676.9 ± 3.3%	72.5 ± 6.9%	63,650.2 ± 3.6%	38,527.8 (−0.16)
crown6	1408.2 ± 4.9%	28,164.6 ± 4.9%	46,644.0 ± 1.9%	60.4 ± 3.0%	54,162.6 ± 4.9%	35,762.9 (−0.23)
crown8	1639.8 ± 1.2%	32,795.8 ± 1.2%	56,956.6 ± 0.9%	57.6 ± 0.3%	63,068.8 ± 1.2%	42,579.7 (−0.25)
notch3	1867.1 ± 6.5%	37,341.2 ± 6.5%	74,028.1 ± 4.9%	50.4 ± 1.6%	71,810.0 ± 6.5%	65,234.5 (−0.12)
notch4	1943.6 ± 5.8%	38,871.5 ± 5.8%	77,731.0 ± 3.5%	50.0 ± 2.3%	74,752.9 ± 5.8%	65,896.2 (−0.15)
notch5	2074.2 ± 3.9%	41,483.7 ± 3.9%	75,516.9 ± 0.7%	54.9 ± 4.6%	79,776.3 ± 3.9%	67,234.5 (−0.11)
notch6	2186.2 ± 12.2%	43,723.9 ± 12.2%	72,117.2 ± 1.0%	60.6 ± 11.2%	84,084.4 ± 12.2%	60,125.8 (−0.17)
notch8	1682.2 ± 9.3%	33,644.3 ± 9.3%	71,107.1 ± 5.0%	47.3 ± 4.3%	64,700.6 ± 9.3%	57,783.2 (−0.19)
notch9	1911.8 ± 0.1%	38,235.5 ± 0.1%	62,601.2 ± 8.4%	61.1 ± 8.5%	73,529.9 ± 0.1%	49,923.5 (−0.20)
notch10	1846.6 ± 5.9%	36,932.8 ± 5.9%	57,724.8 ± 10.6%	64.0 ± 16.3%	71,024.7 ± 5.9%	49,337.8 (−0.15)

Note: Columns PCF sim are uniformly expressed in the format of “simulated value (relative error of simulation with respect to experiment)”.

## Data Availability

The original contributions presented in this study are included in the article. Further inquiries can be directed to the corresponding author.
